# α-Iso-Cubebene Inhibits PDGF-Induced Vascular Smooth Muscle Cell Proliferation by Suppressing Osteopontin Expression

**DOI:** 10.1371/journal.pone.0170699

**Published:** 2017-01-23

**Authors:** Min A. Jang, Seung Jin Lee, Seung Eun Baek, So Youn Park, Young Whan Choi, Chi Dae Kim

**Affiliations:** 1 Department of Pharmacology, School of Medicine, Pusan National University, Gyeongnam, Republic of Korea; 2 Gene & Cell Therapy Research Center for Vessel-associated Diseases, Pusan National University, Gyeongnam, Republic of Korea; 3 College of Pharmacy, Pusan National University, Busan, Republic of Korea; 4 College of Natural Resources & Life Sciences, Pusan National University, Gyeongnam, Republic of Korea; Qatar University College of Health Sciences, QATAR

## Abstract

α-Iso-cubebene (ICB) is a dibenzocyclooctadiene lignin contained in Schisandra chinensis (SC), a well-known medicinal herb that ameliorates cardiovascular symptoms. Thus, we examined the effect of ICB on vascular smooth muscle cell (VSMC) proliferation, a key feature of diverse vascular diseases. When VSMCs primary cultured from rat thoracic aorta were stimulated with PDGF (1–10 ng/ml), cell proliferation and osteopontin (OPN) expression were concomitantly up-regulated, but these effects were attenuated when cells were treated with MPIIIB10, a neutralizing monoclonal antibody for OPN. In aortic tissues exposed to PDGF, sprouting VSMC numbers increased, which was attenuated in tissues from OPN-deficient mice. Furthermore, VSMC proliferation and OPN expression induced by PDGF were attenuated dose-dependently by ICB (10 or 30 μg/ml). Reporter assays conducted using OPN promoter-luciferase constructs showed that the promoter region 538–234 bp of the transcription start site was responsible for transcriptional activity enhancement by PDGF, which was significantly inhibited by ICB. Putative binding sites for AP-1 and C/EBPβ in the indicated promoter region were suggested by TF Search, and increased binding of AP-1 and C/EBPβ in PDGF-treated VSMCs was demonstrated using a ChIP assay. The increased bindings of AP-1 and C/EBPβ into OPN promoter were attenuated by ICB. Moreover, the PDGF-induced expression of OPN was markedly attenuated in VSMCs transfected with siRNA for AP-1 and C/EBPβ. These results indicate that ICB inhibit VSMC proliferation by inhibiting the AP-1 and C/EBPβ signaling pathways and thus downregulating OPN expression.

## Introduction

Vascular smooth muscle cells (VSMCs) are essential regulators of vascular function [1,2]. In healthy arteries, VSMCs are located in the medial vascular layer, where they express contractile proteins that regulate vessel tone and blood flow [3]. However, endoluminal vascular interventional procedures cause stretching of the vessel wall and cell necrosis [4], and subsequently release endogenous molecules activating vascular inflammatory processes [5]. During the vascular inflammatory processes, the recruitment of monocytes to the lesion tissues and subsequent transformation into macrophages concomitant with overproduction of inflammatory cytokines would be major steps [6]. This, in turn, stimulates VSMC proliferation resulting in the development of vascular wall remodeling including atherosclerosis and restenosis after vascular injury [7,8].

Previous studies have demonstrated that OPN levels were elevated in human atherosclerotic plaque [9,10] and neointima after experimental angioplasty [11]. Thus, OPN has been suggested to be implicated in vascular injury responses by increasing extracellular matrix invasion, migration and proliferation of VSMCs [12–14]. Furthermore, OPN was reported to be strongly expressed in a synthetic VSMC phenotype [15], and suggested to be a key factor of the development of vascular remodeling diseases [16,17]. Although the vascular remodeling effects of OPN have aroused considerable research interest [18], little is known of its role in vascular wall remodeling.

*Schisandra chinensis* (SC) has a long history as a medicinal herb and is a traditional component in oriental medicines [19,20]. Several authors have suggested SC may have beneficial regulating effects in patients with cardiovascular diseases, as its aqueous extract induced vasorelaxation in rat thoracic aorta [21,22]. In the previous study, we demonstrated that gomisin A and gomisin J isolated from SC relaxed vascular smooth muscle, suggesting a potential therapeutic role in hypertensive patients [23,24]. Also, Choi et al. [25] reported the antioxidant properties of α-iso-cubebene (ICB), a dibenzocyclooctadiene lignin found in SC, and suggested its potential use to ameliorate the symptoms of cardiovascular disease. However, little is known about the effect of ICB on VSMC proliferation, which is characteristic feature of many vascular diseases.

Under pathological conditions, VSMCs exhibit phenotypic changes characterized by loss of contractility, abnormal proliferation, migration, and matrix secretion [10]. This synthetic phenotype of VSMCs plays an active role in the development of several cardiovascular diseases, including vascular remodeling diseases [26–28]. In view of the known participation of OPN in the progression of vascular remodeling diseases [17,29], we considered that the identification of molecular regulators of OPN expression in VSMCs might be of importance. Accordingly, we undertook this study to determine the relations between ICB and OPN and PDGF-stimulated VSMC proliferation, and to identify the ICB-targeted transcription factors underlying OPN expression in VSMCs.

## Materials and Methods

### Purification of α-iso-cubebene

α-Iso-cubebene (ICB) was purified from dried fruits of *Schisandra chinensis* (SC) as described previously [30]. Briefly, SC (2.5 kg) fruit was dried, and ground to a fine powder, and successively extracted at room temperature with *n*-hexane, chloroform (CHCl_3_), and methanol (MeOH). The hexane extract (308 g) was evaporated in vacuo and chromatographed on a 40 μm silica gel (J.T. Baker, Phillipsburg, NJ, USA) column (100 × 10 cm) using step gradient elution (0%, 5%, and 20% ethyl acetate in hexane and 5% methanol MeOH in CHCl_3_ to obtain 38 fractions). Fraction 1 (KH1PA, 3,689 mg) was separated on a silica gel column (100 × 3.0 cm) using 15% acetone in dichloromethane (CH_2_Cl_2_) to obtain nine fractions, and the second fraction (KH1PAIB, 999 mg) was separated on a silica gel column (100 × 3.0 cm) using 15% acetone in CH_2_Cl_2_ to yield ICB (316 mg). Pure ICB (purity > 99%) was identified by high-performance liquid chromatography on a Phenomenex Luna C18 column (150 × 4.6 mm internal diameter; 5 μm particle size) using an acetonitrile-water-alcohol gradient at a flow rate of 1.0 ml/min.

### Ethics statement and animals

All animal procedures conformed with the Guide for the Care and Use of Laboratory Animals published by the US National Institute of Health (NIH Publication No. 85–23, 2011 revision), and the experimental protocols were approved by the Pusan National University Institutional Animal Care and Use Committee. All genotyping, including that of OPN deficient mice was performed by PCR using a protocol provided by the Jackson Laboratory (Harlan Nossan, Italy). Wild-type (WT) control mice (C57BL/6J) were purchased from Jackson Laboratories.

### Chemicals and antibodies

Platelet derived growth factor (PDGF) was purchased from Sigma (St. Louis, MO), and OPN (sc-21742) and β-actin (sc-47778) antibodies were purchased from Santa Cruz Biotechnology Inc. (Beverly, MA). Horseradish peroxidase (HRP)-conjugated IgG antibody (Santa Cruz Biotechnology Inc.) was used as the secondary antibody. PCR primers were from Bioneer (Seoul). AP-1 (10024-2-AP) antibody was purchased from Proteintech (Proteintech Group, Chicago, USA), and C/EBPβ (ab15049) antibody from Abcam (Cambrige, MA). Restriction enzymes were supplied by Promega (Madison, WI).

AP-1 and C/EBPβ siRNA oligonucleotides were synthesized by Bioneer (Daejeon, Korea). siRNA molecules were transfected into cells using Lipofectamine 2000 siRNA transfection reagent (Invitrogen, Carlsbad, CA), according to the manufacturer's instructions. siRNA sequences against AP-1 and C/EBPβ were as follows: AP-1, ACUGUAGAUUGCUUCUGUA (sence) and UACAGAAGCAAUCUACAGU (antisense); C/EBPβ, GACAAGCUGAGCGACGAGU (sence) and ACUCGUCGCUCAGCUUGUC (antisense).

### Cell culture and MTT assay

Sprague-Dawley rats (Charles River Breeding Laboratories, Kingston, NY, USA) were sacrificed by CO_2_ inhalation, and then primary VSMCs was cultured from thoracic aorta. Briefly, excised aortas were cut into ~1 mm^2^ segments, and placed as explants in a cell culture dish containing DMEM (Gibco BRL, Grand Island, NY) with 10% FBS (Gibco BRL). Cells were maintained in DMEM containing 10% FBS and antibiotic-antimycotic (Gibco BRL) at 37°C.

An MTT assay was used to determine the proliferation rates of VSMCs. Briefly, cells (a total of 1x10^5^ cells) were treated with MTT working solution (EZ-Cytox, Daeil Laboratories, Seoul, Republic of Korea), and incubated at 37°C for 1 hr. OD values of solution was obtained at a wavelength of 450 nm by ELISA. Relative proliferation rates were determined by comparing cells with control cells.

### Western blot analysis

VSMC lysates were prepared in ice-cold lysis buffer, and equal amounts of the protein obtained were separated on 8~10% polyacrylamide gel under reducing conditions, and then transferred to nitrocellulose membranes (Amersham-Pharmacia Biotech, Piscataway, NJ). Membranes were blocked with 5% skim milk in TBST and incubated overnight with primary antibody in 5% skim milk. Blots were washed with TBST, and incubated with HRP-conjugated secondary antibody for 2 hrs. Blots were developed using ECL Western blot detection reagents (Amersham). Membranes were re-blotted with anti-β-actin antibody (Santa Cruz Biotechnology) as an internal control.

### Measurement of mRNA expression

OPN mRNA levels in VSMCs were quantified by RT-PCR using GAPDH mRNA as an internal standard. Total RNA was isolated from cells using Quiazol (Qiagen, Hilden, Germany) and reverse transcribed into cDNA using the Improm-II Reverse Transcription System (Promega). cDNA amplification was performed using primers specific for OPN (forward, 5'-CCGATGAGGCTATCAAGGTC-3'; reverse, 5'-ACTGCTCCAGGCTGTGTGTT-3').

### Preparation of OPN promoter and luciferase assays

A series of constructs of OPN promoter in luciferase expression vector pGL3-basic (Promega) were prepared. The OPN promoter was amplified from genomic DNA using the following PCR primers (forward 5'-AGTGTAGGAAGCAGTCAGTCCTGTCAG-3'; reverse 5'-TACCTTGGCTGGCTTCTCGAGCATGCT-3'), and then cloned into pGL3-basic to generate a pLuc-OPN-2284 construct. Additional deletion constructs lacking distal promoter sequences (denoted pLuc-OPN-538 and pLuc-OPN-234) were prepared by digesting pLuc-OPN-2284 with restriction enzymes (*NheI*, *Sac*1 or *Xho*1).

All plasmids were prepared using the QIAprep spin kit (Qiagen Inc., Hilden, Germany). Cells were transfected with plasmids using Lipofectamin 2000 Transfection Reagent (Zymed Laboratories; Invitrogen), according to the manufacturer's instructions. Cell lysates were prepared using the passive lysis buffer from the Promega assay system (Promega, Madison, WI) and luciferase activity was determined using the dual luciferase reporter assay system (Promega).

### Chromatin immunoprecipitation assay

Chromatin immunoprecipitation (ChIP) analysis was performed using the Sigma ChIP kit (Sigma, Saint Louis, MO) according to the manufacturer's instructions with minor modifications. Briefly, VSMCs were inoculated into a 15-cm dish (8 × 10^7^ cells) and fixed with formaldehyde (1%). Cell pellets were then resuspended in shearing buffer containing protease inhibitors (1 mM AEBSF, 1 mg/ml aprotinin, and 1 mg/ml pepstatin A), sonicated with a Misonixsonicator 3000 (Misonix, Farmingdale, NY, USA), centrifuged, and diluted 10-fold in ChIP dilution buffer. Chromatin samples were incubated at room temperature for 90 minutes with assay well bound AP-1 and C/EBPβ antibody, and hybridized at 65°C for 4 hrs. Released DNA was collected through the GenElute Binding Column G, and immunoprecipitated chromatins were analyzed by PCR using the following OPN gene promoter primers (forward 5'-AGAAGGTCTCACTCTGTTGCCCAT-3'; reverse 5'-AGAATCCTGGAAGAGCATCAGGGA -3'). The cycling parameters were; 63°C for 1 min, 95°C for 30 sec, followed by 40 amplification cycles.

### Statistical analysis

Results were expressed as means ± SEMs. One-way analysis of variance (ANOVA) followed by Turkey’s multiple comparison test or unpaired Student’s t-test were used to determine the significance of differences. Statistical significance was accepted for p value < 0.05.

## Results

### Characteristics of OPN expression in VSMCs stimulated with PDGF

**Fig 1:**
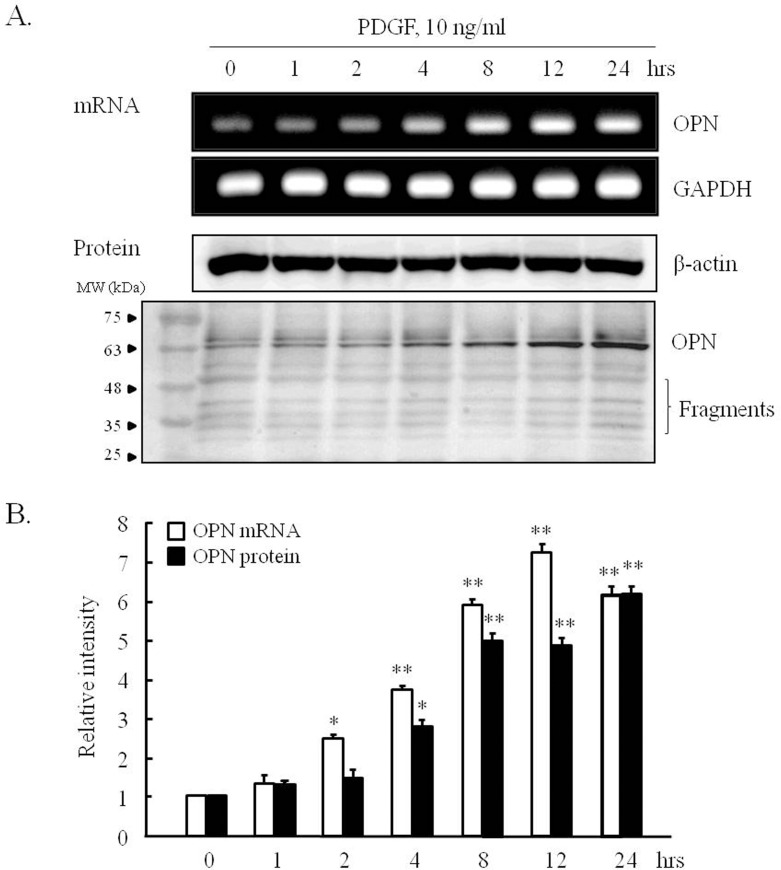
Characteristics of OPN expression in PDGF-stimulated VSMCs. (A) VSMCs were stimulated with 10 ng/ml of PDGF for the indicated times, and OPN mRNA and protein levels were then assessed by RT-PCR and Western blotting. Images are representative of 4–6 independent experiments. MW, molecular weight marker (kDa). (B) Relative intensities of OPN mRNA and protein versus GAPDH and β-actin were quantified. Results were expressed as the means ± SEMs of 4–6 independent experiments. *P<0.05 and **P<0.01 vs. corresponding value at 0 hr.

To investigate the effect of PDGF on OPN expression in VSMCs, cells were stimulated with PDGF (10 ng/ml) for 24 hrs, and OPN mRNA and protein levels were determined by RT-PCR and Western blotting, respectively. OPN mRNA levels in PDGF-treated cells started to increase after 2 hrs of PDGF treatment and continued to increase until 24 hrs (Fig 1A). Likewise, OPN protein levels in cells started to increase from 4 hrs of PDGF treatment (Fig 1B).

### Functional role of OPN in PDGF-induced VSMC proliferation

**Fig 2:**
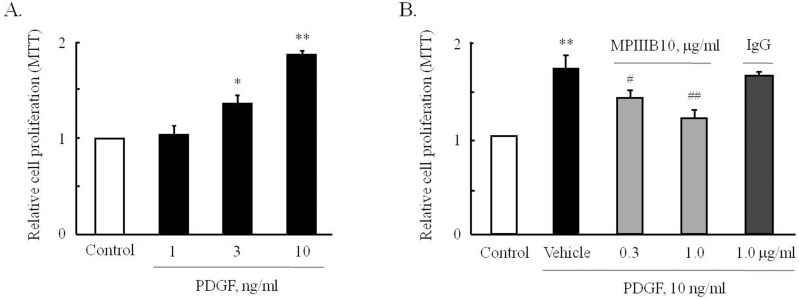
Role of OPN during PDGF-induced VSMC proliferation. (A) Cells were stimulated with the indicated concentrations of PDGF for 48 hrs, and then MTT assays were performed. Relative cell proliferation was expressed as the means ± SEMs of 8 independent experiments. *P<0.05 and **P<0.01 vs. control. (B) Cells were pre-treated with the indicated doses of MPIIIB10 (a neutralizing monoclonal antibody for OPN) or IgG for 1 hr, and cell proliferation was assessed using a MTT assay. Relative cell proliferation was expressed as the means ± SEMs of 4 independent experiments. **P<0.01 vs. control, ^#^*P*<0.05 and ^##^*P<*0.01 *vs*. vehicle.

To investigate the functional role of OPN in PDGF-induced VSMC proliferation, primary cultured VSMCs from rat thoracic aorta were pretreated with MPIIIB10 (a neutralizing monoclonal antibody for OPN), and then cell proliferation was induced by PDGF. MTT assay results showed that PDGF (1–10 ng/ml) dose-dependently increased VSMC proliferation (Fig 2A). The increased VSMC proliferation induced by 10 ng/ml of PDGF was significantly and dose-dependently attenuated by pretreating MPIIIB10 at 0.3 and 1.0 μg/ml, but not by pretreating IgG at 1.0 μg/ml (Fig 2B).

**Fig 3:**
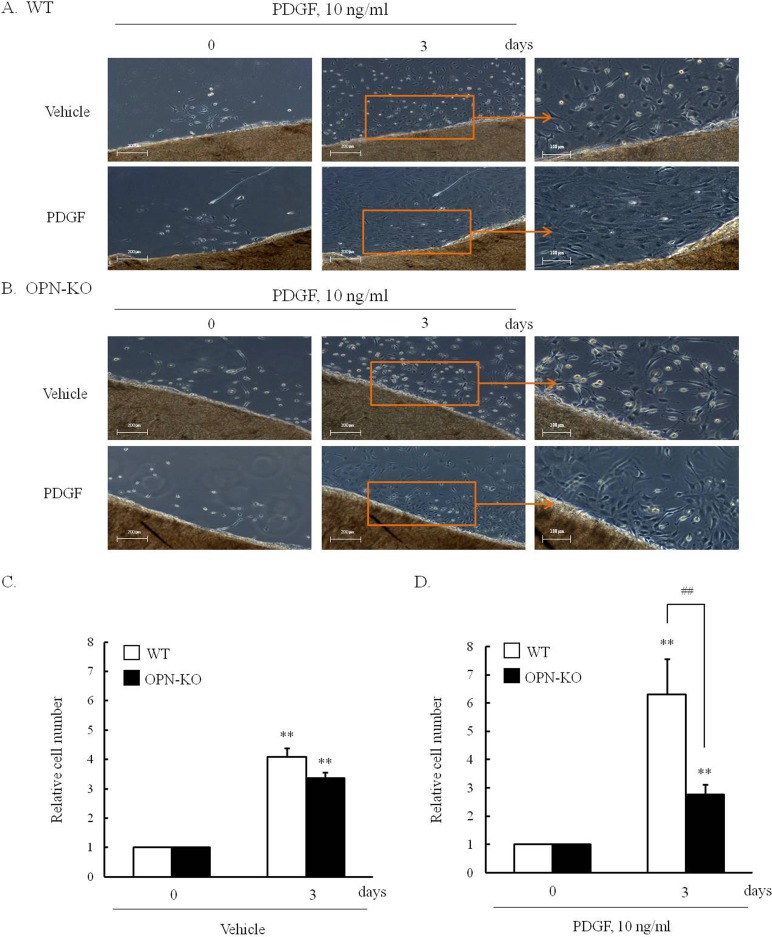
Comparison of explant cultures of VSMCs from the aortic tissues of WT and OPN-KO mice. (A) and (B), Explant cultures of the aortic tissues of WT and OPN-KO mice for 3 days were performed with or without PDGF. Sprouting VSMCs were shown in the photographs. Microscope images are representative of 5 independent experiments. (C) and (D), Sprouting cells from the aortic tissues were counted and relative numbers to 0 day were expressed as the means ± SEMs of 5 independent experiments. **P<0.01 vs. corresponding value at 0 day, ^##^*P*<0.05 vs. WT.

As shown in Fig 3, numbers of sprouting VSMCs was increased when aortic tissues of WT or OPN-KO mice were incubated in culture medium containing 0.5% FBS, however, the difference between groups were not observed. In aortic tissues of WT mice exposed to PDGF for 3 days, numbers of sprouting VSMCs were markedly increased, which was significantly attenuated in tissues of OPN-KO mice. These results suggested that OPN plays a pivotal role in PDGF-induced VSMC proliferation.

### Effect of ICB on PDGF-induced VSMC proliferation and OPN expression

**Fig 4:**
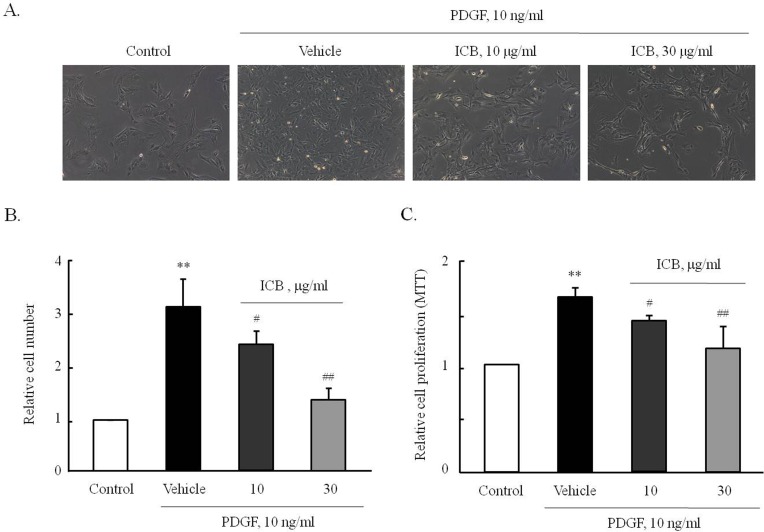
Effects of ICB on PDGF-induced VSMC proliferation. (A) Cells were pre-treated with the indicated doses of ICB (10 and 30 μg/ml) for 4 hrs and then stimulated with 10 ng/ml of PDGF for 48 hrs. Microscope images are representative of 8 independent experiments. (B) Cells in culture plate were counted and relative cell numbers to control were expressed as the means ± SEMs of 8 independent experiments. **P<0.01 vs. control, ^#^*P*<0.05 and ^##^*P<*0.01 *vs*. vehicle. (C) Cell proliferation was assessed using a MTT assay. Relative cell proliferation was expressed as the means ± SEMs of 8 independent experiments. **P<0.01 vs. control, ^#^*P*<0.05 and ^##^*P<*0.01 *vs*. vehicle.

To determine the effects of ICB on PDGF-induced VSMC proliferation, cells were pretreated with 10 or 30 μg/ml of ICB for 2 hrs, and then stimulated with 10 ng/ml of PDGF for 48 hrs. Microscopic images of VSMCs cultured on 12 well plates (Fig 4A) and the cell proliferation assays (Fig 4B and 4C) showed that PDGF at a concentration of 10 ng/ml significantly increased VSMC proliferation, and this was markedly and dose-dependently attenuated in cells pretreated with ICB.

**Fig 5:**
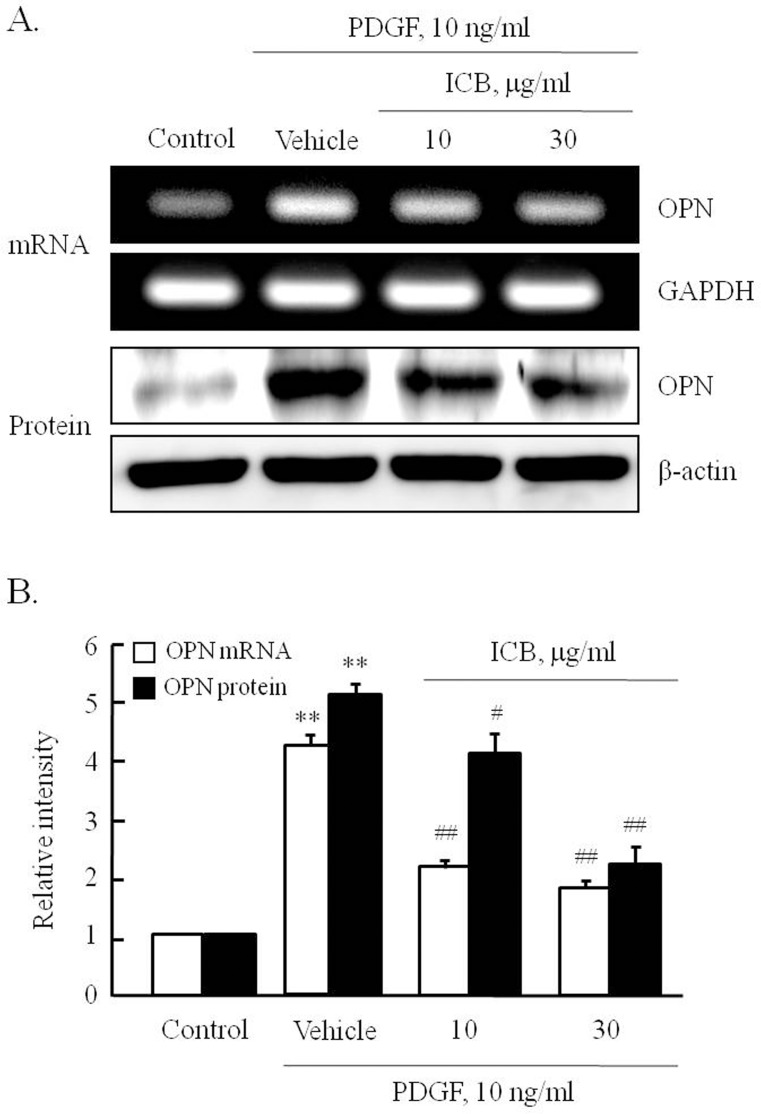
Effects of ICB on PDGF-induced OPN expression in VSMCs. (A) Cells were pre-treated with the indicated doses of ICB for 4 hrs, and then stimulated with 10 ng/ml of PDGF for 12 hrs. OPN mRNA and protein levels were assessed by RT-PCR and Western blotting. Images are representative of 5–7 independent experiments. (B) Relative intensities of OPN mRNA and protein versus GAPDH and β-actin were expressed as the means ± SEMs of 5–7 independent experiments. **P<0.01 vs. corresponding control, ^#^*P*<0.05 and ^##^*P<*0.01 *vs*. corresponding vehicle.

To determine the effects of ICB on PDGF-induced OPN expression in VSMCs, cells were stimulated with 10 ng/ml of PDGF for 12 hrs in the presence of ICB at 10 or 30 μg/ml. As shown in Fig 5, OPN mRNA and protein levels markedly increased in PDGF-stimulated cells, and these increases were significantly and concentration-dependently reduced by ICB pretreatment. These results suggest OPN expression in PDGF-stimulated VSMC is modulated by ICB at the transcriptional levels.

### Identification of the ICB-targeted transcription factors mediating OPN expression in VSMCs

**Fig 6:**
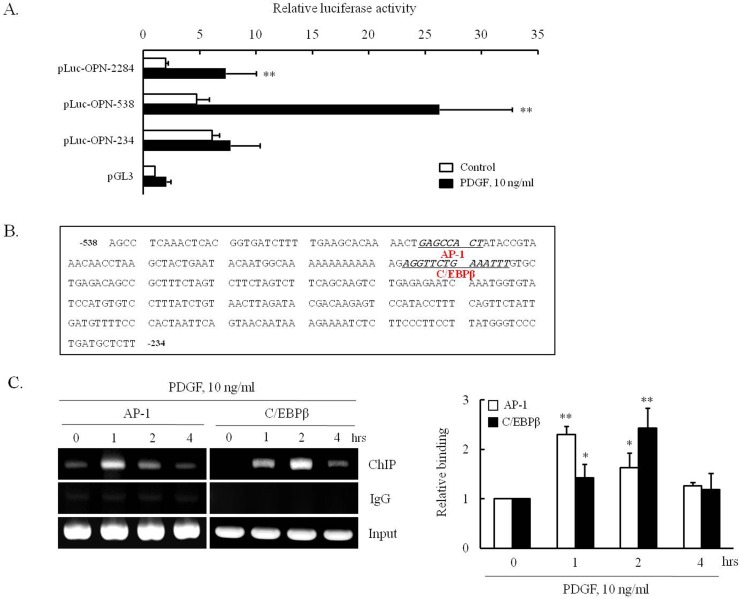
Identification of transcription factors involved in PDGF-induced OPN expression in VSMCs. (A) Cells were transiently transfected with various promoter constructs or an empty luciferase vector (pGL3) for 24 hrs, and then stimulated with PDGF (10 ng/ml) for 4 hrs. Relative luciferase activities were presented as the means ± SEMs of 12 independent experiments. **P<0.01 vs. non-treated control. (B) Nucleotide sequence of the -538 ~ -234 promoter region of the OPN gene. The transcription factor binding sites were identified using TFSearch software. The sequences of potential binding sites for AP-1 and C/EBPβ in pLuc-OPN-538 were underlined. (C) The bindings of AP-1 and C/EBPβ in PDGF-treated VSMCs were assessed using a ChIP assay. IgG was used as negative control. The images are representative of 4 independent experiments. Quantitative results were expressed as the means ± SEMs of 4 independent experiments. *P<0.05 and **P<0.01 vs. value at 0 hr.

To identify the responsible *cis*-acting elements in OPN promotor, three OPN luciferase constructs were established (pLuc-OPN-2284, -538 and -234). As shown in Fig 6A, relative luciferase activities were measured after transiently transfecting these three constructs into VSMCs. The luciferase reporter activity of pLuc-OPN-2284 and pLuc-OPN-538 in VSMCs exposed to 10 ng/ml of PDGF was about 3- and 5-folds higher than that in control. In contrast, this increase in PDGF-induced luciferase activity was abolished in cells transfected with the pLuc-OPN-234 construct (Fig 6A). These results suggest that the -538 ~ -234 region of OPN promoter is responsible for PDGF-induced OPN transcription in VSMCs. Putative binding sites for AP-1 and C/EBPβ in this region were suggested by a TF Search (Fig 6B), and the increased binding of AP-1 and C/EBPβ in PDGF-treated VSMC was demonstrated by a ChIP assay (Fig 6C).

**Fig 7:**
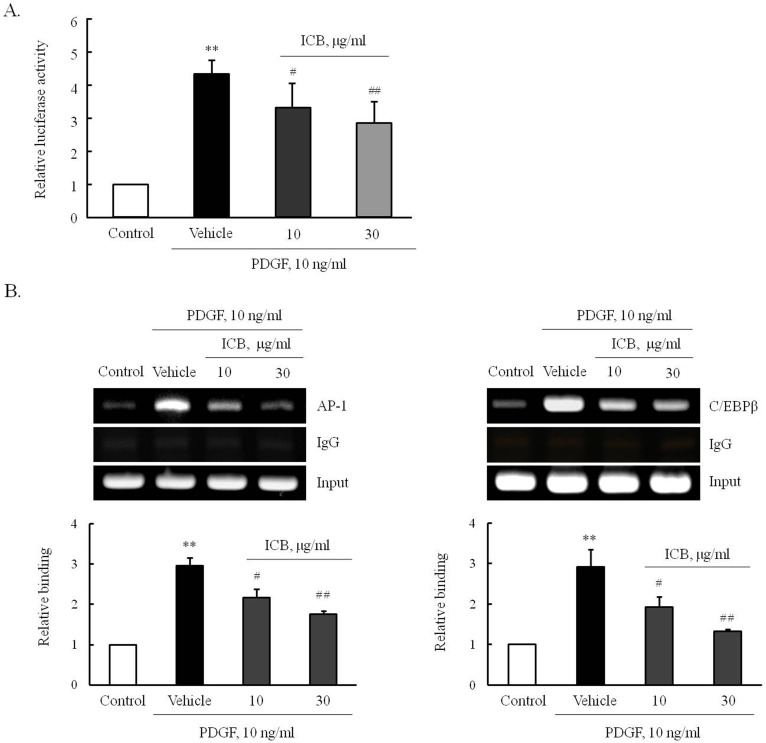
Identification of ICB-targeted transcription factors in VSMCs stimulated with PDGF. (A) Cells were transiently tansfected with pLuc-OPN-538 constructs for 24 hrs, pretreated with the indicated doses of ICB, and then stimulated with PDGF (10 ng/ml) for 4 hrs. Relative luciferase activities were presented as the means ± SEM of 8 independent experiments. **P<0.01 vs. control, ^#^*P*<0.05 and ^##^*P*<0.01 vs. vehicle. (B) The effects of ICB on the bindings of active AP-1 and C/EBPβ were assessed using a ChIP assay. IgG was used as negative control. Images are representative of 4 independent experiments. Quantitative results were expressed as the means ± SEMs of 4 independent experiments. **P<0.01 vs. corresponding control, ^#^*P*<0.05 and ^##^*P*<0.01 vs. corresponding vehicle.

**Fig 8:**
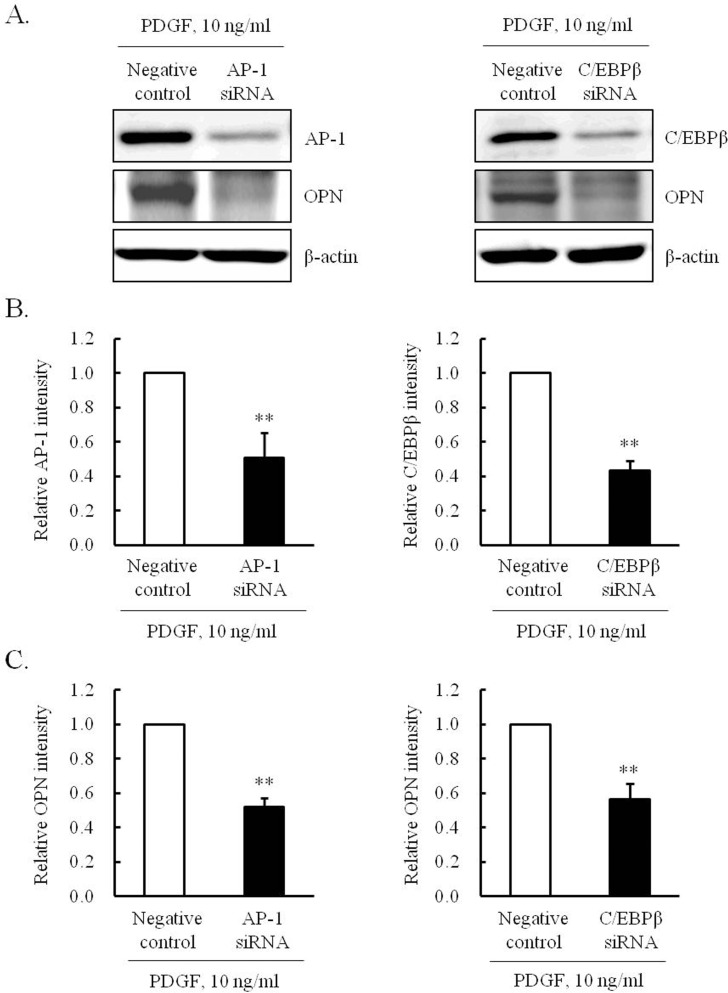
Involvement of AP-1 and C/EBPβ in PDGF-induced OPN expression in VSMCs. (A) Cells were transfected with 100 pmol/L of AP-1 siRNA or C/EBPβ siRNA for 24 hrs, and then stimulated with 10 ng/ml of PDGF for 12 hrs. The expression of AP-1/CEBPβ and OPN in siRNA-transfected cells were assessed by Western blotting. Images are representative of 4 independent experiments. (B and C) Quantitative results were expressed as the means ± SEMs of 4 independent experiments. **P<0.01 vs. corresponding negative control (NC).

To determine the effect of ICB on PDGF-induced OPN transcription, VSMCs were transfected by pLuc-OPN-538 for 24 hrs, pretreated with ICB, and then stimulated with PDGF. As shown in Fig 7A, PDGF-enhanced luciferase activity in cells transfected with pLuc-OPN-538 was markedly attenuated in cells pretreated with ICB. Moreover, the increased binding of AP-1 and C/EBPβ into OPN promoter in PDGF-stimulated cells was attenuated by ICB pretreatment, suggesting a potential role of AP-1 and C/EBPβ in the PDGF-induced expression of OPN in VSMCs. In VSMCs transfected with siRNA for AP-1 and C/EBPβ, the PDGF-induced expression of OPN was also markedly attenuated (Fig 8), demonstrating a central role for AP-1 and C/EBPβ in OPN expression in PDGF-stimulated cells.

## Discussion

This study importantly shows PDGF increases OPN expression in VSMCs and subsequently enhances VSMC proliferation. In addition, ICB was found to attenuate VSMC proliferation and OPN expression induced by PDGF. Increased bindings between AP-1 or C/EBPβ and OPN promoter in PDGF-stimulated cells were demonstrated by a ChIP assay, and these bindings were attenuated by ICB. These results suggested that ICB attenuated PDGF-induced VSMC proliferation by inhibiting the AP-1 and C/EBPβ signaling pathways and consequently down-regulating OPN expression in VSMCs.

A number of studies have demonstrated that OPN is important for VSMC proliferation and migration into intima [13,31]. In particular, OPN levels were found to be elevated in atherosclerotic plaque and neointima after experimental angioplasty or carotid artery stenting, and OPN-induced VSMC proliferation has been implicated in atherosclerosis and vascular injury response [32]. However, comparatively little is known about the role played by OPN in PDGF-induced VSMC proliferation, although OPN is considered as one of the most potent mitogen of VSMCs and is known to play a central role in the pathogenesis of various vascular disorders [33,34]. Thus, we investigated both the characteristics of OPN expression in PDGF-stimulated VSMCs and the involvement of OPN in PDGF-induced VSMC proliferation. When primary cultured VSMCs from rat thoracic aortas were stimulated with PDGF, cell proliferation was increased in association with upregulated expression of OPN. PDGF-induced VSMC proliferation was attenuated in cells treated with MPIIIB10, an antibody for OPN. Moreover, in aortic tissues exposed to PDGF, numbers of sprouting VSMCs increased, and this was attenuated in the tissues of OPN-deficient mice, indicating a pivotal role of OPN in VSMC proliferation.

The proliferation of VSMCs plays an important role in the development and progression of cardiovascular diseases, including atherosclerosis [35,36], and thus, the modulation of growth factor-stimulated VSMC proliferation has important therapeutic implications [37,38]. Reportedly, *Schisandra chinensis* (SC) has long been used as a tonic, sedative, astringent, anti-aging agent, and to treat cardiovascular symptoms in Korea, China and Japan [39,40]. The major bioactive components of SC fruits are lignans such as ICB, schizandrins and gomisins, such as, gomisin J, N and A [25,41]. In our previous study, hexane extracts of SC were found to cause vasorelaxation in endothelium (ED)-intact vasculature and in ED-denuded rat thoracic aortas [22]. The relaxant effect of SC extracts on ED-intact vasculature was more prominent than that on ED-denuded aorta [22], which suggested the vascular relaxation evoked by SC extracts was mediated mainly by an ED-dependent nitric oxide (NO) pathway.

Therefore, in the present study, we investigated the effects of ICB on VSMC proliferation and OPN expression after stimulating cells with PDGF. Our findings indicated that ICB, a novel small molecule purified from SC, efficiently and dose-dependently inhibited VSMC proliferation. In addition, ICB potently inhibited OPN expression via transcriptional inhibition in PDGF-stimulated VSMCs. Based on our results and those of other studies in which OPN expression was found to be regulated by several mechanisms, including gene expression at the transcriptional and translational levels [42,43]. The present study demonstrated that PDGF stimulation increased OPN protein and mRNA levels, and concomitantly enhanced OPN promoter activity, suggesting the regulation of OPN expression by PDGF at the transcriptional level.

In the hope of identifying a potential strategy for treating vascular remodeling diseases, we also investigated the inhibitory effect of ICB on PDGF-induced OPN expression at the transcriptional and translational levels. To identify the regulatory element in OPN promoter responsible for gene transcription, a 2,284 kb 5' fragment of the open reading frame of OPN was cloned by PCR. In our study, the luciferase reporter activity of pLuc-OPN-538 in VSMCs exposed to 10 ng/ml of PDGF was about 5-fold higher than the control, whereas this increase was not observed in cells transfected with the pLuc-OPN-234 construct. These results suggest that the -538 ~ -234 region of OPN promoter is the *cis*-acting element responsible for PDGF-induced OPN transcription in VSMC. Using the sequence motif search of TFSearch software (http://mbs.cbrc.jp/research/db/TFSEARCH.html), putative transcription factor binding sites for AP-1 and C/EBPβ were identified between -538 bp and -234 bp relative to the transcriptional initiation site in OPN promoter, and subsequently, ChIP assay demonstrated the increased binding of AP-1 and C/EBPβ in PDGF-treated VSMCs. Moreover, the PDGF-induced increase in OPN expression was also markedly attenuated in VSMCs transfected with siRNA for AP-1 and C/EBPβ. These results suggested that the transcription factors, AP-1 and C/EBPβ, regulate OPN expression in PDGF-stimulated VSMCs.

To determine the effect of ICB on PDGF-induced OPN transcription, VSMCs were transfected by the pLuc-OPN-538 construct for 24 hrs. When VSMCs were stimulated with PDGF in the presence of ICB, PDGF-enhanced luciferase activity in cells transfected with pLuc-OPN-538 was markedly attenuated, suggesting a potential inhibition of OPN transcription induced by PDGF. In addition, the increased bindings of AP-1 and C/EBPβ into OPN promoter in PDGF-stimulated cells was attenuated by ICB, indicating that AP-1 and C/EBPβ were major ICB-targeted transcription factors involved in OPN expression in VSMCs.

Summarizing, our experimental results suggested ICB inhibits PDGF-induced VSMC proliferation by inhibiting the AP-1 and C/EBPβ signaling pathways and consequently down-regulating OPN expression. We believe the findings of the present study shed light on the mechanism responsible for the anti-atherogenic activity of ICB.

## Abbreviations

PDGF: platelet-derived growth factor
ICB: alpha-iso-cubebene
VSMC: vascular smooth muscle cell
OPN: osteopontin
AP-1: activator protein 1
C/EBPβ: CCAAT/enhancer-binding protein beta
ChIP: chromatin immunoprecipitation
MTT: 3-(4,5-dimethylthiazol-2-yl)-2,5-diphenyltetrazolium bromide
SC: schisandra chinensis
ED: endothelium
